# Multi-Layer Magnetic Shields Based on Fe-Based Nanocrystalline and Co-Based Amorphous Ribbons

**DOI:** 10.3390/ma19101986

**Published:** 2026-05-11

**Authors:** Yanfeng Liang, Benchang Liu, Haoran Ma, Lining Pan, Aina He, Yaqiang Dong, Qikui Man, Jiawei Li

**Affiliations:** 1 School of Materials Science and Chemical Engineering, Ningbo University, Ningbo 315211, China; liangyanfeng@nimte.ac.cn (Y.L.); liubenchang@nimte.ac.cn (B.L.); 2 Zhejiang Key Laboratory of Magnetic Materials and Applications, Ningbo Institute of Materials Technology & Engineering, Ningbo 315201, China; panlining2013@nimte.ac.cn (L.P.); hean@nimte.ac.cn (A.H.); dongyq@nimte.ac.cn (Y.D.); manqk@nimte.ac.cn (Q.M.); 3 University of Chinese Academy of Sciences, Beijing 100049, China

**Keywords:** magnetic shielding, nanocrystalline, amorphous alloys, finite element analysis

## Abstract

We constructed a multi-layer composite magnetic shield composed of Fe-based nanocrystalline (FN) and Co-based amorphous (CA) ribbons, and focused on the influence of the number of layers and their arrangement on the shielding effectiveness (SE). Finite element analysis (FEA) and layer-by-layer inversion calculations were performed to analyze the attenuation process of the magnetic field between shield layers. Increasing the number of shield layers improves the maximum value of SE (*SE_max_*) and significantly broadens the working range (*WWR*). In a weak magnetic field, CA exhibits higher shielding performance, whereas FN is better in a strong magnetic field. The FN/FN/CA combination (FN is closer to the field source) exhibits an *SE_max_* of up to 51.7 dB within a *WWR* of 674.3 A/m, and demonstrates a 14.4% improvement in *SE* compared to FN/FN/FN combination across the entire tested magnetic field range. Finally, a gradient layering design is proposed that enables each layer to operate within its optimal permeability range, thereby improving the overall SE and broadening the effective working magnetic field range.

## 1. Introduction

Magnetic shielding is a pivotal technique for mitigating ambient magnetic interference, including geomagnetic fields and environmental fluctuations, to establish ultra-low, highly homogeneous, and low-noise magnetic environments [1,2,3]. The demand for high-efficiency magnetic shielding spans various frontiers, including fundamental physics [4,5], biomedicine [6,7,8], space research [9], and industrial applications [10]. Shielding apparatuses play a critical role in ensuring the accuracy and sensitivity of magnetic detection. Effective shielding necessitates high-permeability materials [11,12,13] that concentrate magnetic flux lines within the shielding medium, thereby achieving substantial field attenuation [14]. Conventional soft magnetic materials (permalloy, ferrites, and nanocrystalline alloys) [10,15,16] exhibit commendable shielding effects.

Multi-layer structures are considered an effective way to improve magnetic shielding efficiency, primarily because each layer shunts the magnetic field, which greatly enhances the overall capacity to attenuate the magnetic field [11,17]. For single-material structures, DC shielding effectiveness was improved by employing split permalloy layers or multi-layered ribbon-wound coils [18,19]. Xu et al. [20] developed an equivalent electro-magnetic parameter model for anisotropic nanocrystalline materials, enabling precise calculation of magnetic noise and quantifying the influence of stacking factors on low-frequency noise (1–30 Hz). Furthermore, Shen et al. [21] fabricated Fe-based nanocrystalline ribbons with a permeability of 64,000 via a three-step annealing process; the resulting ten-layer shield achieved a noise floor as low as 0.84 fT Hz^−1^/^2^. Regarding heterogeneous composite configurations, Xu et al. [20] utilized a three-layer permalloy and one-layer aluminum structure to achieve a static shielding coefficient of 104. Xu et al. [17] proposed a nanocrystalline/permalloy laminated structure that yielded a static SE of 55.5 dB at a thickness of 2.3 mm. Yamazaki et al. [22,23] demonstrated via FEA that shielding efficiency is maximized when an aluminum layer is positioned centrally within permalloy stacks. Kamata et al. [24] and Xu et al. [25] further optimized shielding coefficients by investigating the coupling effects of thickness, spacing, and material combinations. Notably, Lee et al. [26] pointed out that materials with high saturation induction are more stable in high-field regions, whereas those with high initial permeability offer superior efficiency in low-field zones.

Co-based amorphous alloys [27,28,29] are promising candidates for addressing the precision limits of ultra-weak magnetic measurements due to their ultra-high permeability, minimal power loss, and mechanical flexibility [21,30]. Magnetic shielding effectiveness is inherently constrained by the nonlinear nature of magnetic permeability and flux closure characteristics [31,32,33]. As the external field intensity escalates, the shielding coefficient fluctuates due to these nonlinear effects, potentially leading to magnetic saturation and a consequent degradation in shielding efficacy. Previous studies have indicated a consistent trend between the SE-H response and the μ-H profile—both exhibiting an initial increase followed by a decrease—suggesting an intrinsic physical correlation [34]. However, systematic research on the correlation between the composite shielding characteristics and the permeability profiles of individual constituent materials remains scarce. In particular, the regulatory effect of Co-based composites on shielding efficiency in low-field environments has not yet been elucidated [30].

Building upon this background, inspired by the definitions of *SE_max_* and *H_max_* by Tatiana Zubar et al. [35], we introduce the Width of Working Range (*WWR*) as a novel metric for the quantitative assessment of shielding stability across broad magnetic field intervals. This approach extends the assessment from single-point peak evaluation to interval efficiency evaluation, enabling quantitative characterization of the “broad-field adaptability” of multi-layer structures. Utilizing Fe-based nanocrystalline (FN) ribbons and 6025Z Co-based amorphous (CA) ribbons [36], we constructed multi-layer cylindrical shields with varying arrangement (1~3 layers). The complete SE(H) curves were systematically measured under continuous external fields to extract *SE_max_*, *H_max_*, and WWR. Furthermore, FEA was employed to extract axial field distributions, unveiling the critical physical mechanisms underlying the enhancement of *H_max_* with increasing layer counts and the regulatory patterns of composite arrangement sequences on wide-field shielding efficiency.

## 2. Materials and Methods

**Figure 1:**
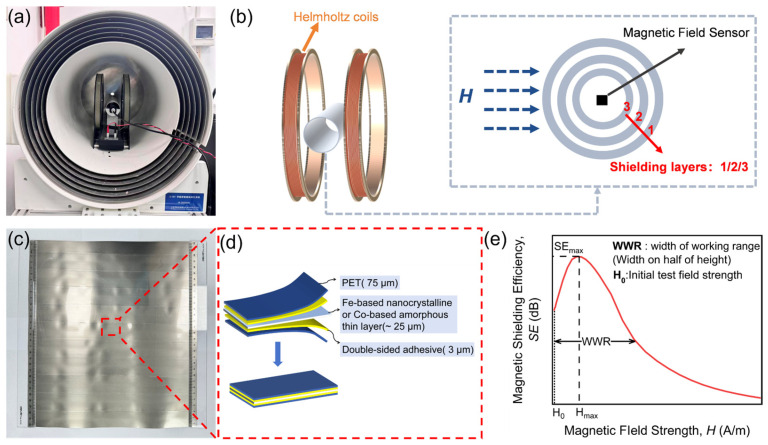
(**a**) Experimental setup of magnetic field shielding performance test. (**b**) The schematic diagram of the test system. (**c**) Optical photograph and (**d**) structural diagram of one single shield layer. (**e**) Schematic representation of the parameters to quantify the magnetic shielding performance.

The experimental setup of the magnetic field shielding performance test is presented in Figure 1a. All experimental measurements are conducted within a D-30T zero-field generation system (CH-Magnetoelectricity Technology Co., Ltd., Beijing, China) to ensure environmental isolation. Figure 1b gives the schematic diagram of the test system. This system uses Helmholtz coils (inside diameter: 300 mm, spacing between coil centers: 150 mm, CH-Magnetoelectricity Technology Co., Ltd., Beijing, China) to generate a uniform one-dimensional magnetic field. The coil geometry satisfies the Helmholtz condition, making the magnetic field in the central region highly uniform. Controlled by a DC power supply, a current of 0.1 A corresponds to a magnetic field of approximately 113 μT. The shields are coiled into cylinders (70 mm diameter, 300 mm length) and positioned in the central zone of the Helmholtz coils, perpendicular to the magnetic field. The strength of magnetic field before (*H*_0_) and after (*H_s_*) shielding is measured using a magnetic field sensor (Mag-13MS1000 fluxgate meter, Bartington Instruments, Witney, UK). This sensor features a measurement range of ±1000 μT, a noise floor of 6 pT/√Hz at 1 Hz, and a factory-calibrated scale error of ±0.5%. Prior to testing, the sensor was placed inside the D-30T zero-field chamber for zero calibration to eliminate any residual offset. The sensor was connected to an external monitoring unit for magnetic field measurement. The naming convention for multiple overlapping shielding layers is presented. The magnetic shields are prepared from Fe-based nanocrystalline (FN) (Ningbo B Plus New Material Technology Co., Ltd., Ningbo, China) and home-made Co-based amorphous (CA) ribbons. One single shield layer consists of more than 10 ribbons forming a uniform plane, which are laminated with 3 μm double-sided adhesive with a Polyethylene Terephthalate (PET) film glued onto both sides (see Figure 1c,d) [21].

The shielding efficiency (SE) of the magnetic shield is evaluated in this study using the equation [35]:

(1) $$SE=20{log}_{10}\frac{H_{0}}{H_{s}}$$

Figure 1e shows schematically how the parameters are determined to quantify the shielding performance of magnetic shields [35]. The maximum value of *SE* (*SE_max_*) and the strength of magnetic field (*H_max_*), which corresponds to the maximum, are obtained by measuring *SE* as a function of magnetic field strength. The Width of Working Range (*WWR*) is obtained as the width from the initial test field (where the corresponding *SE* value is greater than half of the peak-value *SE_max_*) to the field strength corresponding to half of *SE_max_* in the high-field region to describe the peak shape of the SE-H curve and quantify the width of the shield’s working range.

The phase structures of the ribbons are characterized by X-ray diffraction (XRD, Bruker D8 Advance, Bruker Corporation, Billerica, MA, USA) with Cu Kα radiation. The magnetization curve and hysteresis loop are measured using a B-H loop tracer (MATS-3000S, Hunan Lianzhong Co. Ltd., Loudi, China) under a maximum applied field of 800 A/m. The test specimens present as annular with inner diameter of 20 mm and outer diameter of 30 mm.

To analyze the attenuation process of the magnetic field between shield layers, finite element simulations were performed using COMSOL Multiphysics software (v. 6.1, COMSOL AB, Stockholm, Sweden) based on magnetostatic theory [3,37,38,39]. The experimental cylindrical shield (diameter 70 mm, length 300 mm, ribbon thickness 0.025 mm) was simplified into a two-dimensional axisymmetric model to improve computational efficiency while maintaining accuracy [33], with all layers assumed to be continuous and homogeneous. Since the applied field is a DC field, the skin effect was neglected. An adaptive meshing strategy with local refinement was adopted, with a minimum element size of 0.01 mm in the shielding region (ensuring 3–5 elements across the thickness), gradually increasing to 5 mm in the transition region and up to 20 mm in the far field. The computational domain was extended to 500 mm × 500 mm to reduce boundary effects [40]. For boundary conditions, an axisymmetric condition was applied along the central axis. A uniform external magnetic field (0–650 A/m, 14 points) was imposed at the outer surface to simulate the Helmholtz coil field, while the outer boundary was set as magnetic insulation. The experimentally measured nonlinear permeability μ(H) of Co-based amorphous (CA) and Fe-based nanocrystalline (FN) ribbons was used as input. Parametric sweeps were conducted to obtain magnetic field distributions, and the shielding effectiveness (*SE*) was calculated accordingly to characterize the shielding performance of multi-layer structures.

## 3. Results

### 3.1. Structure and Magnetic Properties of Shielding Materials

**Figure 2:**
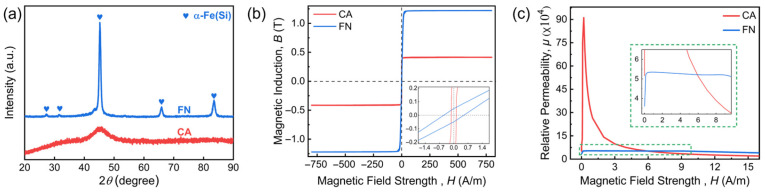
(**a**) XRD patterns, (**b**) hysteresis loops and (**c**) permeability curves of Fe-based nanocrystalline (FN) and *Co*-based amorphous (CA) ribbons.

Figure 2a presents the XRD patterns for FN and CA ribbons. CA exhibits a characteristic broad diffraction hump with no observable crystalline peaks, confirming the amorphous nature. In contrast, FN display a series of sharp peaks corresponding to the a-Fe(Si) phase, indicating the nanocrystalline structure [21].

The hysteresis loops of FN and CA ribbons are shown in Figure 2b. Compared to FN, CA exhibits a lower saturation magnetic induction (*B_s_*) and lower coercivity (*H_c_*, see the insert in Figure 2b). Figure 2c presents the magnetic permeability of FN and CA ribbons as a function of the magnetic field. The permeability, expressed as the *B*/*H* ratio, is converted by the initial magnetization curves. The permeability of both ribbons first increases with the strength of the magnetic field, then decreases, and finally gradually becomes a stable value. The initial (*μ_i_*) and maximum (*μ_max_*) permeability of CA are much higher than those of the FN. The magnetic field (*H_μ_*) corresponding to *μ_max_* is smaller for CA than for FN [36]. Furthermore, the permeability of CA decays faster than that of FN: although CA exhibits a higher permeability in weak magnetic fields, its permeability begins to fall below that of FN at a stronger field strength. The magnetic property parameters of both ribbons are listed in Table 1. The magnetic shielding performance depends on the permeability of the shield. We will show below that the difference in the permeability would exert a significant influence on the magnetic shielding performance of the two types of ribbons.

### 3.2. Magnetic Shielding Performance

**Figure 3:**
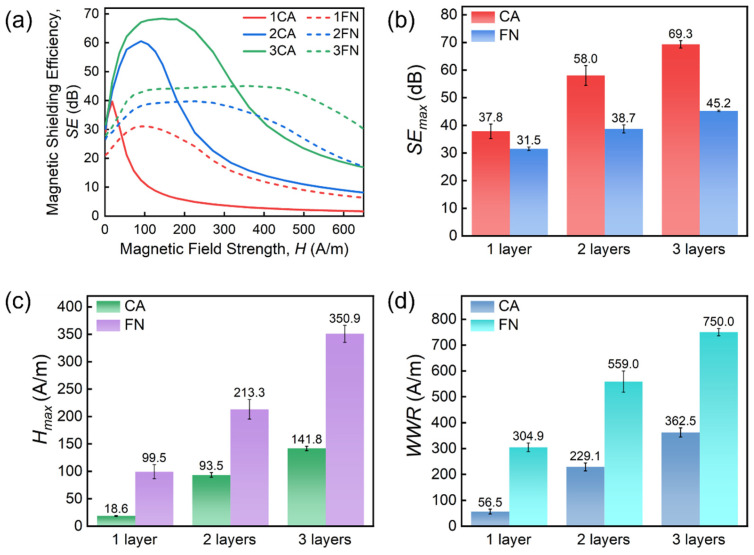
(**a**) Change in *SE* with change in the number of shield layers. Change in (**b**) *SE_max_*, (**c**) *H_max_* and (**d**) *WWR* with the number of shield layers.

Figure 3a shows the influence of the change in the number of shield layers on SE of CA and FN, respectively [41,42]. All samples exhibit a similar trend in SE as a function of magnetic field strength, consistent with the variation in their permeability. The extraction of key parameters to quantify the shielding performance follows the methodology described in Figure 1e, and the results are given in Figure 3b–d. We see that *SE_max_*, *H_max_* and WWR all increase with an increase in the number of shield layers. In Figure 3b, *SE_max_* of a single-layer CA reaches 37.8 dB, while that of two-layer and three-layer CA increases to 58.0 dB and 69.3 dB, respectively. *SE_max_* for the single-, two- and three-layer FN are 31.5, 38.7 and 45.2 dB, respectively. Both types of ribbons exhibit superior magnetic field shielding performance. In addition, CA has a higher *SE_max_* than FN with the same layer number, due to the higher *μ_max_* of the former. However, the *μ_max_* of CA appears at a weaker magnetic field strength, resulting in a lower *H_max_* value than FN (see Figure 3c). The *H_max_* of the single-, two- and three-layer types are 18.6, 93.5 and 141.8 A/m for CA, and 99.5, 213.3 and 350.9 A/m for FN. The *WWR* of CA is also lower than that of FN (see Figure 3d). This is because the permeability of CA decays more quickly, resulting in a narrower working range. In conclusion, under weak magnetic fields, CA yields the highest shielding effect, while FN provides a better shielding effect under strong magnetic fields.

**Figure 4:**
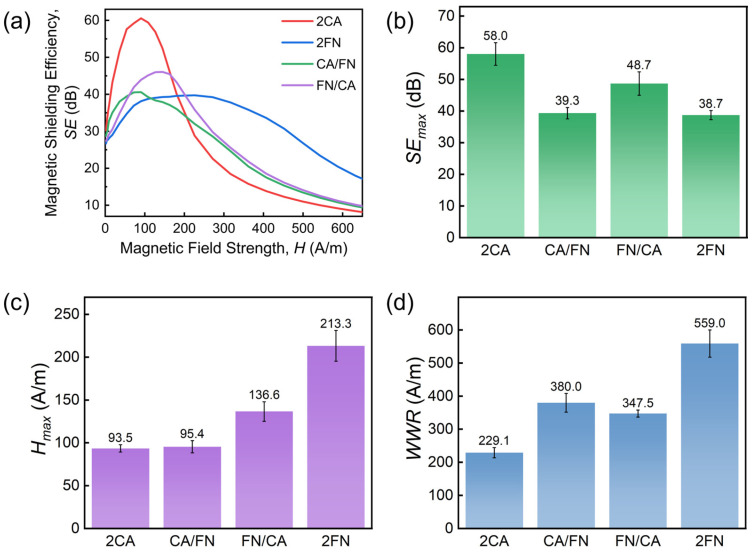
(**a**) Change in *SE* of two-layer shield with change in the lamination order. Change in (**b**) *SE_max_*, (**c**) *H_max_* and (**d**) *WWR* with the lamination order of shield layer.

Different lamination orders of the multi-layer shield would have different shielding effects on the magnetic field. Figure 4a shows changes in *SE* in shielding with two layers of alloy ribbons. Two layers with different materials produce a different SE evolution than two layers of the same material. The performance levels of both the CA/FN (CA is closer to the field source) and the FN/CA pairs are between those of the 2CA and the 2FN pairs (see Figure 4b–d). The *WWR* of CA/FN and FN/CA are similar, but the latter shows a higher *SE_max_* and *H_max_*. Only under small-scale weak magnetic fields does the former have better shielding performance. In summary, for double-layer shields, 2CA and 2FN, made of the same materials, exhibit better magnetic shielding performance under weak and high fields, respectively.

**Figure 5:**
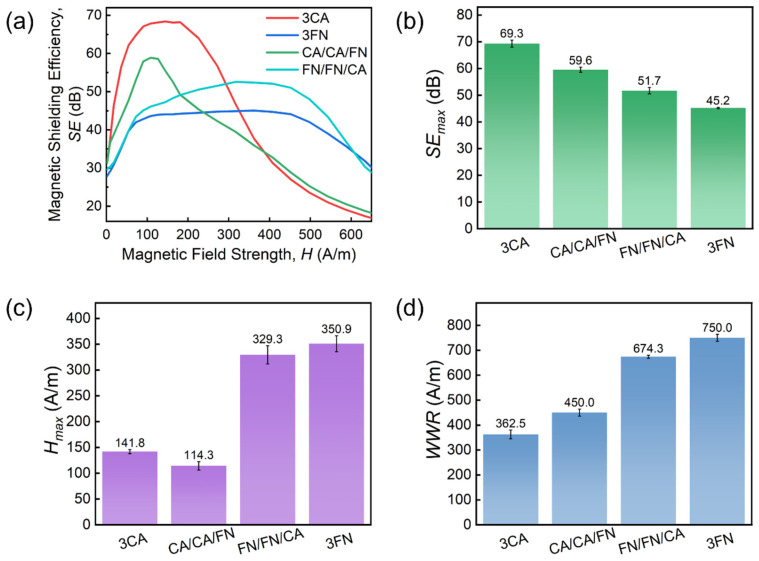
(**a**) Change in *SE* of three-layer shield with change in the lamination order. Change in (**b**) *SE_max_*, (**c**) *H_max_* and (**d**) *WWR* with the lamination order of shield layer.

Changes in SE in shielding with three layers of alloy ribbons are further evaluated, as shown in Figure 5a. Here, we only show representative data for CA/CA/FN and FN/FN/CA; data for other lamination orders are provided in the Supplementary Figure S1. Similarly, the performance levels of both the CA/CA/FN and FN/FN/CA pairs are between those of the 3CA and 3FN pairs (see Figure 5b–d). It is worth noting that the shielding performance of FN/FN/CA is superior to that of 3FN across almost the entire tested magnetic field range. Consequently, under high field conditions, FN/FN/CA becomes a better choice for magnetic shielding compared to 3FN, while 3CA still exhibits the best magnetic shielding effect under low field conditions.

**Figure 6:**
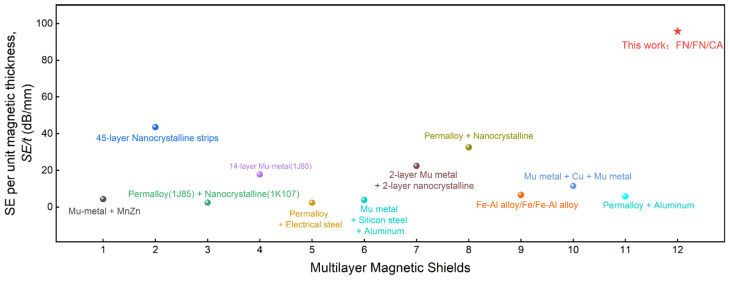
Normalized DC shielding performance per unit magnetic thickness across various shielding combinations from the literature. The horizontal axis labels 1–12 correspond to the combinations listed in Table 2.

Table 2 summarizes the shielding performance of the magnetic shields in this work and those reported in the literature. The comparison focuses on the material system, total magnetic material thickness and the reported *SE*. A noteworthy observation is that most existing multi-layer shields achieve high *SE* at the expense of substantially thicker material layers (typically on the order of millimeters), whereas the FN/FN/CA combination in this work attains a competitive *SE* of 51.7 dB with a total layer thickness of only approximately 0.5 mm. To provide a more intuitive comparison of shielding performance normalized by material usage, Figure 6 presents the normalized SE per unit layer thickness (dB/mm) for the combinations listed in Table 2. The FN/FN/CA combination proposed in this work achieves the highest normalized *SE* among all composite designs, demonstrating its exceptional shielding performance within an ultra-thin magnetic layer stack.

## 4. Discussion

**Figure 7:**
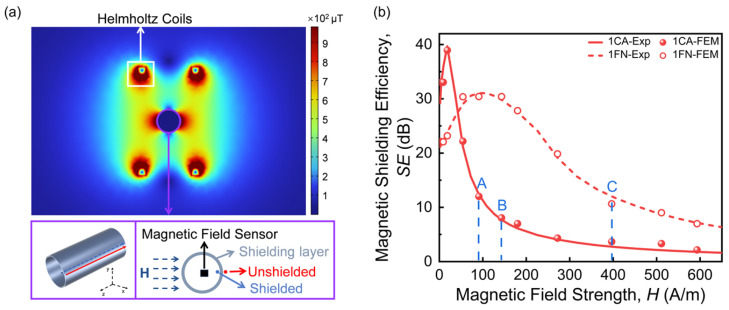
(**a**) Simulated flux densities of single-layer CA shield at a magnetic field of 522 A/m. (**b**) Simulated and experimental SE of single-layer CA and FN.

To reveal more details on the magnetic shielding mechanism of the various shielding configurations, FEM was used to simulate *SE* of the proposed shields. The magnetic shielding model consisted of Helmholtz coils and a single-layer cylindrical shield was established, both of which have the same dimensions as those used in the experimental setup. The shield layers were modeled as continuous and homogeneous shells, whereas the actual structure consists of discrete ribbons laminated with PET films. This simplification neglects the additional reluctance introduced by ribbon overlaps and microscopic air gaps, which may cause the simulated *SE* to be slightly higher than measured values in some regions. Similar homogeneous approximations have been employed in FEM studies of laminated nanocrystalline shields [15], where the close agreement between the simulation and experiment (relative error < 10%) supports the validity of this approach. Additionally, the skin effect was neglected and the experimentally measured *μ*(H) curves were used under a quasi-static approximation, which captures the nonlinear saturation behavior but disregards magnetic hysteresis and remanence. For the DC fields considered in this work, the quasi-static approximation is justified. However, the model is not directly applicable to transient or AC field conditions. Figure 7a gives an example of the simulated flux densities of single-layer CA at a magnetic field of 522 A/m. As shown in Figure 7b, the simulated *SE* was plotted in a graph and superimposed with experimental results. To quantitatively evaluate the deviation between the simulated and experimental data after shielding, we calculated the relative error using the following equation:

(2) $$\delta =\frac{\left|{SE}_{sim}-{SE}_{exp}\right|}{{SE}_{exp}}\times 100\%$$

where *SE_sim_* and *SE_exp_* are the simulated and experimental *SE*, respectively. The average relative error for 1CA is only 6.5%, while for 1FN, the average error is also as low as 4.3%, indicating good agreement between theoretical simulations and experimental tests.

### 4.1. Mechanisms of Single-Material Multi-Layer Shielding

**Figure 8:**
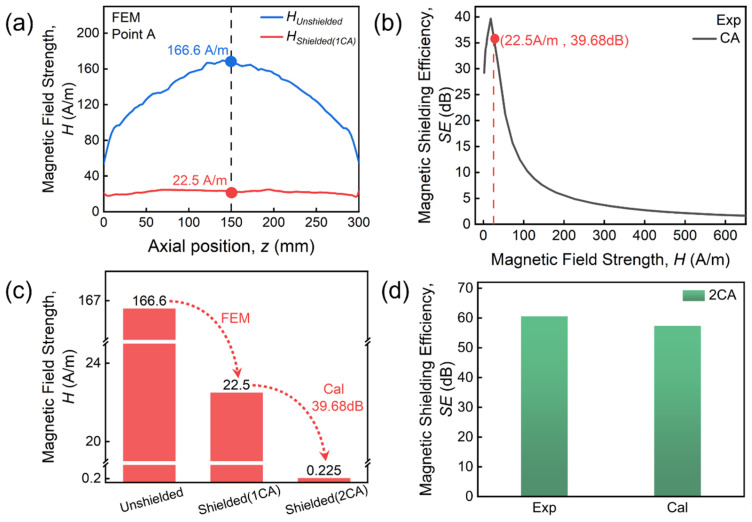
(**a**) Simulated radial magnetic field distributions inside and outside the single-layer CA shield under a magnetic field of 90.42 A/m. (**b**) Measured SE at a specific field of 22.5 A/m. (**c**) Simulated and calculated magnetic fields after single- and two-layer CA shielding. (**d**) Comparison between calculated and experimental measured SE for 2CA shielding.

For multi-layer CA shields, the increase in layer number increases the *SE_max_* value (Figure 3). At the same time, *H_max_* is also significantly increased, revealing that the multi-layer structure not only improves the shielding effect but also broadens the effective shielding magnetic field range. To address the physical origin of such results, the magnetic field attenuation characteristics of the multi-layer CA shield are analyzed. Firstly, the radial magnetic field distributions are extracted at a distance of 1 mm from both the interior and exterior surfaces of the single-layer shield, as illustrated in Figure 7a. Figure 8a shows the extracted data for 1CA under a magnetic field of 90.42 A/m (*H_max_* for 2CA, point A indicated in Figure 7b). We see a significant effect of converging magnetic field lines on the surface of the shield. The peak value of the magnetic field before shielding (*H_Unshield_*) reaches 166.6 A/m, significantly higher than the applied external magnetic field. After passing through single-layer CA shielding, the magnetic field (*H_Shielded_*) attenuates to 22.5 A/m. For a two-layer CA shield, this magnetic field is the same as *H_Unshield_* of the inner CA. Secondly, *H_Shielded_* of the inner CA is calculated based on the experimental SE data. As shown in Figure 8b, SE is 39.68 dB at the magnetic field of 22.5 A/m, corresponding to the high SE range of 1CA. Therefore, the calculated *H_Shielde__d_* is 0.225 A/m, i.e., the magnetic field after passing through 2CA shielding under an external magnetic field of 90.42 A/m. Finally, the calculated SE for 2CA is 60.56 dB, very close to the experimental value (57.36 dB, see Figure 8d), with a relative error of only 6% between them.

**Figure 9:**
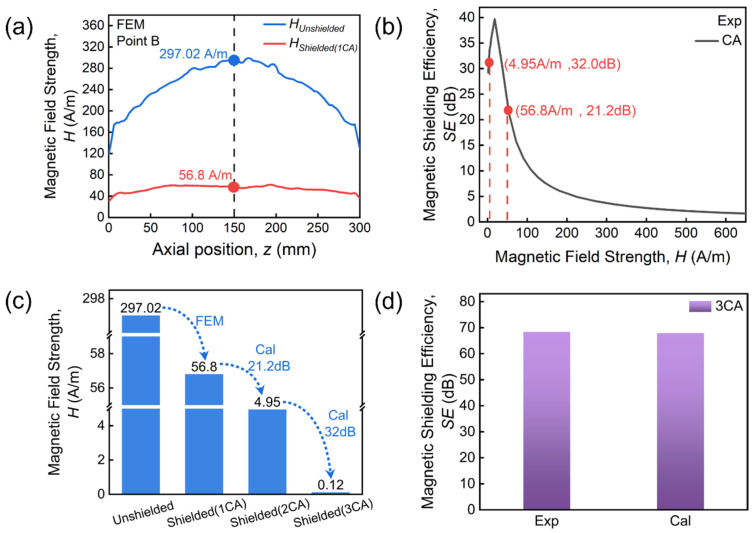
(**a**) Simulated radial magnetic field distributions inside and outside the single-layer CA shield under a magnetic field of 144.66 A/m. (**b**) Measured SE at specific fields of 56.8 and 4.95 A/m. (**c**) Simulated and calculated magnetic fields after single-, two- and three-layer CA shielding. (**d**) Comparison between calculated and experimental measured SE for 3CA shielding.

We further consider the case of 3CA following the methodology described above. Firstly, the radial distributions of both *H_Unshield_* and *H_Shielded_* are extracted in Figure 9a for 1CA under a magnetic field of 144.66 A/m (*H_max_* for 3CA, point B indicated in Figure 7b). Now *H_Unshield_* reaches a peak value of 297.02 A/m and attenuates to 56.8 A/m after passing through single-layer CA shielding. Secondly, *H_Shielded_* of the second- and third-layer CA are calculated based on the experimental SE data (Figure 9b), and the results given in Figure 9c. Notably, the magnetic field values of *H_Shielded_* (1CA) and *H_Shielded_* (2CA), i.e., *H_Unshield_* of the second- and third-layer CA, are both located in the high SE region of 1CA. The calculated *H_Shielded_* (3CA) is 0.12 A/m, i.e., the magnetic field after passing through 3CA shielding under an external magnetic field of 144.66 A/m. Finally, the calculated SE for 3CA is 67.9 dB, close to the experimental value (68.37 dB, see Figure 9d), with a relative error of only 1% between them. The physical basis of multi-layer FN shields is similar to the case for multi-layer CA described here.

Based on the above discussion, we conclude that with increasing layers, the multi-layer shield forms a cascading attenuation structure for the magnetic field. The outer layer first interacts with the strong external magnetic field, significantly reducing its intensity, so that the magnetic field transmitted to the inner layers is progressively weakened and falls within the range of relatively high permeability of each layer. Due to the nonlinear dependence of permeability on magnetic field strength, a single-layer shield can only achieve high *SE* within a limited low-field range. In contrast, the multi-layer structure enables step-like attenuation and redistribution of the magnetic field, allowing each layer to operate within its optimal permeability region, thereby achieving synergistic enhancement of the shielding effect. Consequently, the overall SE increases with the number of layers, leading to a higher *SE_max_*, a shift in *H_max_* toward higher field strengths and a broader working field range.

### 4.2. Mechanisms of Composite Multi-Layer Shielding

**Figure 10:**
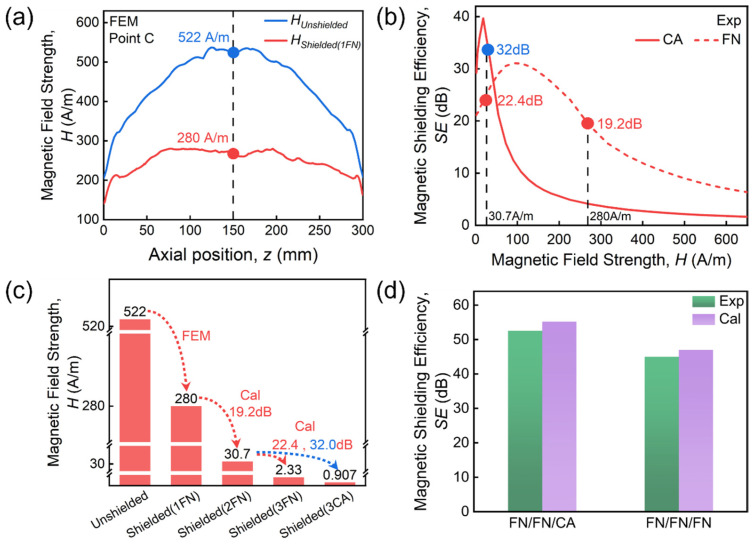
(**a**) Simulated radial magnetic field distributions inside and outside the single-layer CA shield under a magnetic field of 397 A/m. (**b**) Measured *SE* at specific fields. (**c**) Simulated and calculated magnetic fields after 1FN, 2FN, 3FN and FN/FN/CA shielding. (**d**) Comparison between calculated and experimental measured *SE* for FN/FN/CA and 3FN shielding.

We will finish this section with a discussion of different SE values for FN/FN/CA and 3FN (Figure 5). Similarly, the radial distributions of both *H_Unshield_* and *H_Shielded_* of 1FN are extracted in Figure 10a under a magnetic field of 397 A/m (*H_max_* for FN/FN/CA, point C indicated in Figure 7b). *H_Unshield_* reaches a peak value of 522 A/m and attenuates to 280 A/m after passing through single-layer FN shielding. Then, *H_Shielded_* of the second-layer FN is calculated based on the experimental SE data (Figure 10b), and is found to be 30.7 A/m (Figure 10c). We found that such *H_Unshielded_* of the third layer, made of different materials, corresponds to different values of SE: for the third-layer FN, SE is 22.4 dB; SE becomes 32 dB for the third-layer CA. The calculated *H_Shielded_* (3FN) after 3FN shielding is 2.33 A/m, while *H_Shielded_* (FN/FN/CA) after FN/FN/CA shielding is further reduced to 0.907 A/m. Finally, the calculated SE values for 3FN and FN/FN/CA are consistent with experimental results (see Figure 10d), with a relative error of only 4.4% and 5% for 3FN and FN/FN/CA, respectively. Such enhanced magnetic shielding performance achieved by combining materials with different permeability characteristics has been reported previously. Lee et al. [26] found that compared to single-material two-layer shielding, the GO/PC composite exhibits significantly improved *SE*, maintaining excellent performance across a wide magnetic field range (PC has a high permeability in weak fields, while GO exhibits higher permeability under strong fields). A nanocrystalline composite structure with a radially increasing permeability gradient (from inner to outer layers) was reported, which achieves superior magnetic shielding effectiveness [15]. Here, by combining finite element simulation with layer-by-layer inversion calculations, we elucidate the specific attenuation process of the magnetic field within the multi-layer structure. This approach not only deepens the understanding of the shielding mechanisms of multi-layer composite structures against magnetic fields but also provides theoretical guidance for selecting material parameters and layering sequences when designing multi-layer shielding structures for specific magnetic field scenarios.

In conclusion, the three-layer FN/FN/CA exhibits higher *SE* than 3FN across the entire tested magnetic field range, thanks to the complementary magnetic response of the multi-layer shielding. Under strong external magnetic fields, the outer 2FN initially significantly attenuates the magnetic field, reducing it to a lower level while it reaches the innermost layer. At such a low magnetic field, the layer of CA, which possesses superior shielding performance in weak magnetic fields, operates closer to its optimal permeability range and thus provides more effective attenuation than FN under the same conditions. Therefore, the synergistic combination of FN layers for initial attenuation and a CA layer optimized for weak-field shielding enables the FN/FN/CA structure to achieve higher *SE* over the entire magnetic field range than 3FN. This finding provides a guideline for the design of multi-layer magnetic shielding structures: rather than simply increasing the number of identical layers, it is more effective to rationally combine materials with different magnetic field response characteristics to achieve synergistic shielding. Specifically, outer layers should use materials (e.g., FN) with good attenuation capability under high magnetic fields to reduce the initial magnetic field strength, while inner layers should employ materials (e.g., CA) with higher permeability and shielding effectiveness under weak magnetic fields to maximize the attenuation of the already reduced field. Such a gradient layering design enables each layer to operate within its optimal permeability range, thereby improving overall shielding effectiveness and broadening the effective operating magnetic field range.

## 5. Conclusions

By employing Fe-based nanocrystalline (FN) and Co-based amorphous (CA) ribbons, we report on the construction of a multi-layer composite magnetic shielding structure with a varying number and arrangement of shield layers. The attenuation process of the magnetic field between shield layers is analyzed by finite element analysis (FEA) and layer-by-layer inversion calculations to explore shielding mechanisms. The key conclusions of this study are as follows:

- For single-material multi-layer shielding structures, as the number of layers increases from one to three, the *SE* of CA improves from 37.8 dB to 69.3 dB with the corresponding *WWR* broadening from 56.5 A/m to 362.5 A/m; for FN, the SE rises from 31.5 dB to 45.2 dB and its WWR expands from 304.9 A/m to 750 A/m. FN is effective at shielding at higher applied magnetic fields, while CA is applicable at lower fields.
- For two-layer shielding structures, the performance levels of FN/CA (FN is closer to the field source) and CA/FN combinations are between those of FN/FN and CA/CA combinations. For three-layer shielding structures, FN/FN/CA achieves an *SE_max_* of 51.7 dB within a *WWR* of 674.3 A/m, and demonstrates a 14.4% improvement in SE compared to 3FN across almost the entire tested magnetic field range. Such enhanced shielding performance of FN/FN/CA is owing to the synergistic combination of outer FN layers for initial attenuation of strong external magnetic fields and a CA inner layer optimized for the reduced field shielding.

Our findings provide a guidance for the design of high-performance magnetic shielding structures. Moreover, the effective shielding of magnetic fields achieved by multi-layer shields composed of Fe-based nanocrystalline and Co-based amorphous ribbons highlights their potential for practical applications.

## Figures and Tables

**Table 1:** Magnetic properties of FN and CA.

Materials	*μ_i_* (×10^4^)	*μ_max_* (×10^4^)	*H_max_* (A/m)	B_s_ (T)	*H_c_* (A/m)
CA	24.78	91.30	0.58	0.39	0.15
FN	3.84	5.44	1.34	1.25	0.60

**Table 2:** Comparison of DC shielding effectiveness of representative multi-layer and hybrid magnetic shields.

Number	Materials	Thickness (mm)	*SE* (dB)	Source
1	Mu-metal + MnZn ferrite	13.5	58.3	[10]
2	45-layer Nanocrystalline strips	1.1	47.0	[15]
3	Permalloy (1J85) + Nanocrystalline (1K107)	2.3	55.5	[17]
4	14-layer Mu-metal (1J85)	4.0	71.1	[18]
5	Permalloy + Electrical steel	0.7	24.4	[26]
6	Mu metal + Silicon steel + Aluminum	15.0	57.3	[39]
7	2-layer Mu metal + 2-layer nanocrystalline	4.0	89.5	[42]
8	Permalloy + Nanocrystalline	2.8	91.1	[43]
9	Fe-Al alloy/Fe/Fe-Al alloy	7.0	46.4	[44]
10	Mu metal + Cu + Mu metal	7.0	80.3	[45]
11	Permalloy + Aluminum	10.0	57.4	[46]
12	This work: FN/FN/CA	0.5	51.7	

## Data Availability

The original contributions presented in this study are included in the article/Supplementary Material. Further inquiries can be directed to the corresponding authors.

## Supplementary Materials

The following supporting information can be downloaded at: https://www.mdpi.com/article/10.3390/ma19101986/s1, Figure S1: (a) Change in *SE* of three-layer shield with change in the lamination order. Change in (b) *SE_max_*, (c) *H_max_* and (d) *WWR* with the lamination order of shield layer.

## Abbreviations

The following abbreviations are used in this manuscript:

CA	Co-based amorphous ribbons
FN	Fe-based nanocrystalline ribbons
SE	Shielding efficiency
*SE_max_*	The maximum value of SE
*H_max_*	Magnetic field strength corresponding to the *SE_max_*
WWR	The Width of Working Range
