# Supplementary material for: Multi-Layer Magnetic Shields Based on Fe-Based Nanocrystalline and Co-Based Amorphous Ribbons
Source: Materials (Basel). 2026 May 11;19(10):1986. doi: 10.3390/ma19101986 (PMC13208922; doi:10.3390/ma19101986)
Supplement: Supplementary file 1 [file materials-19-01986-s001.zip › materials-4254651-supplementary.pdf]

Supplementary Materials

Multi-layer magnetic shields based on Fe-based nanocrystalline and Co-based amorphous ribbons

…..

Yanfeng Liang[1,2], Benchang Liu[1,2], Haoran Ma[2,*], Lining Pan[2], Aina He[2,3], Yaqiang Dong[2,3], Qikui Man[2,3], Jiawei Li[2,3,*]

1 School of Materials Science and Chemical Engineering, Ningbo University, Ningbo, Zhejiang 315211, China

2 Zhejiang Key Laboratory of Magnetic Materials and Applications, Ningbo Institute of Materials Technology & Engineering, Ningbo, Zhejiang 315201, China

3 University of Chinese Academy of Sciences, Beijing 100049, China

* Correspondence: mahaoran@nimte.ac.cn; lijw@nimte.ac.cn

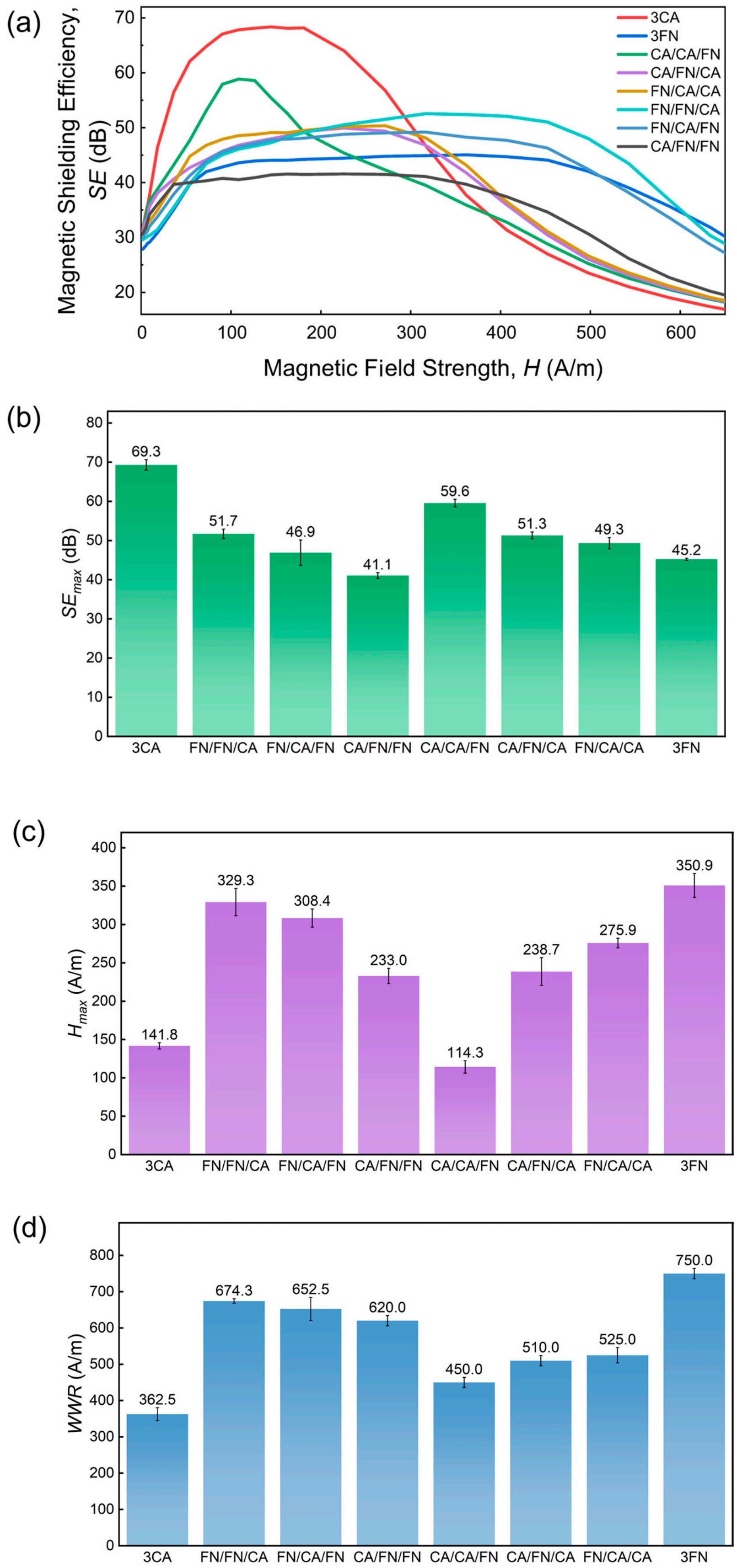

Figure S1. (a) Change in *SE* of three-layer shield with change in the lamination order. Change in (b) SE_{max}, (c) H_{max} and (d) *WWR* with the lamination order of shield layer.
